# Enhancement of Human Epidermal Cell Defense against UVB Damage by Fermentation of *Passiflora edulis* Sims Peel with *Saccharomyces cerevisiae*

**DOI:** 10.3390/nu15030501

**Published:** 2023-01-18

**Authors:** Jiaxuan Fang, Qianru Sun, Ziwen Wang, Zixin Song, Jiman Geng, Changtao Wang, Meng Li, Dongdong Wang

**Affiliations:** College of Chemistry and Materials Engineering, Beijing Technology and Business University, Beijing 100048, China

**Keywords:** *Passiflora edulis* Sims peel, resource utilization, photodamage, skin barrier, inflammation, fermentation technology

## Abstract

The processing of *Passiflora edulis* Sims results in large amounts of wasted peel resources and environmental pollution. In order to improve the utilisation of natural plant resources and economic benefits, this study uses *Saccharomyces cerevisiae* to ferment *Passiflora edulis* Sims peel to obtain *Passiflora edulis* Sims peel fermentation broth (PF). The content of active substances in unfermented *Passiflora edulis* Sims peel water extract (PW) and PF is then determined, as well as their in vitro antioxidant capacity. The protective effects of PF and PW on UVB-induced skin inflammation and skin barrier damage in human immortalised epidermal keratinocytes (HaCaT) cells (including cell viability, ROS, HO-1, NQO1, IL-1β, IL-8, TNF-α, KLK-7, FLG, AQP3 and Caspase 14 levels) are investigated. Studies have shown that PF enhances the content of active substances more effectively compared to PW, showing a superior ability to scavenge free radical scavenging and antioxidants. PW and PF can effectively scavenge excess intracellular ROS, reduce the cellular secretion of pro-inflammatory factors, regulate the content of skin barrier-related proteins and possibly respond to UVB-induced cell damage by inhibiting the activation of the PI3K/AKT/mTOR signalling pathway. Studies have shown that both PW and PF are safe and non-irritating, with PF exploiting the efficacy of *Passiflora edulis* Sims peel more significantly, providing a superior process for the utilisation of *Passiflora edulis* Sims waste. At the same time, PF can be developed and used as a functional protective agent against ultraviolet damage to the skin, thereby increasing the value of the use of *Passiflora edulis* Sims waste.

## 1. Introduction

*Passiflora edulis* Sims, also known as egg fruit and Brazil fruit, belongs to Passiflora, an herbaceous vine native to Brazil and now widely grown in the tropical and subtropical regions of the world. Current research on *Passiflora edulis* Sims focuses on the analysis and application of its juice, but *Passiflora edulis* Sims peel accounts for 50% of its total weight, so there are obvious problems such as resource waste in the application process.

*Passiflora edulis* Sims peel is rich in flavonoids, polyphenols, dietary fibre, protein and other functional components required by the human body [1,2], with excellent anti-inflammatory, antioxidant, blood pressure and lipid-lowering effects [3,4]. Studies have demonstrated that *Passiflora edulis* Sims peel polysaccharides can effectively inhibit the secretion of pro-inflammatory factors and down-regulate mRNA expression in RAW 264.7 macrophages induced by lipopolysaccharide (LPS) damage [5]. In addition, hundreds of phenolic substances were identified by HPLC-MS/MS analysis (quasi-targeted metabolomics) in *Passiflora edulis* Sims peel, which effectively inhibit α-glucosidase activity and possess excellent free radical scavenging and total antioxidant capacity [1]. Extensive research should be carried out on *Passiflora edulis* Sims peel to enhance its value-added and resource utilisation.

Various methods of extracting *Passiflora edulis* Sims peel are currently available, including traditional acid extraction, new enzyme assisted extraction, supercritical CO_2_ extraction, ultrasonic assisted extraction, ultrasonic cell grinder extraction, microwave assisted extraction, microbial fermentation extraction and so on [6,7]. In the industry, traditional extraction methods use a large number of organic reagents. The high cost of supercritical CO_2_ and enzyme assisted extraction is an inevitable obstacle [8]. Notably, microbial fermentation is a safe green extraction method with mild conditions, short extraction time, high economic efficiency and suitability for large-scale production.

Microbial fermentation is a biotransformation method that has been used in the study of many active ingredients. It can convert large molecular weight active substances into small molecular weight active substances [9] and use various biological enzymes secreted by microorganisms in the process of growth and metabolism to help release active substances from within plant cell walls, thereby improving the efficacy of plant active substances [10], effectively reducing substances with toxic side effects in plants [11] and reducing irritation. *Saccharomyces cerevisiae* is a commonly used strain in microbial fermentation, and studies have shown that vine tea fermented with yeast has a greater antioxidant capacity, in some cases exceeding 100 µg/mL of vitamin C, and it can inhibit intracellular tyrosinase activity and melanin synthesis. The zebrafish tail docking inflammation model was used to demonstrate that vine tea fermentation broth exhibited better anti-inflammatory effects with a moderate increase in concentration [12]. Studies have shown that *Saccharomyces cerevisiae* fermentation can alter the structural characteristics and enhance the in vitro antioxidant activity of wheat bran polysaccharides [13].

Although research on *Passiflora edulis* Sims peels is emerging, no attention has been paid to fermented *Passiflora edulis* Sims peel and skin inflammation and skin barrier damage caused by UVB radiation. Specifically, no studies have investigated the protective effects of fermented *Passiflora edulis* Sims peel (PF) on UV-irradiation-induced immortalised keratin-forming cells (HaCaT) in humans.

Ultraviolet radiation is one of the most common and widespread factors affecting skin health, and its effects on skin inflammation and the skin barrier have been the focus of research in recent years. Studies have shown that acute damage from UVB irradiation can contribute to the production of excess reactive oxygen species (ROS) in skin cells. Cell survival requires a certain level of ROS, but the excessive accumulation of ROS can cause mitochondrial dysfunction or even cell death [14] and trigger oxidative stress in humans, which promotes the upregulation of skin inflammatory signalling factors [15], leading to skin inflammation. It also affects the normal functioning of the skin barrier and results in dry, red and flaky skin, leading to an imbalance in skin homeostasis [16,17]. As exposure to ultraviolet radiation is inevitable, there is an urgent need to find more ways to protect the skin and reduce the inflammatory response and barrier damage caused by ultraviolet radiation.

**Figure 1:**
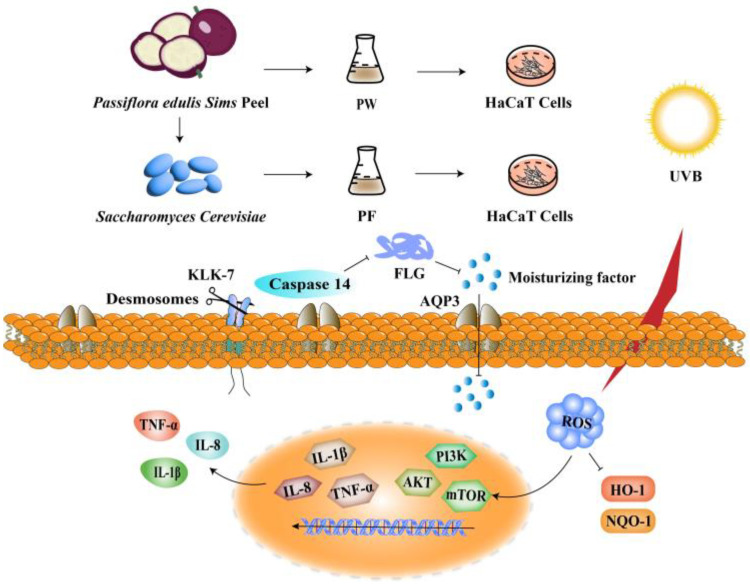
Protective effects of PF and PW on HaCaT damaged by UVB.

In this study, as shown in Figure 1, *Saccharomyces cerevisiae* was used to ferment waste *Passiflora edulis* Sims peel and establish a UVB-induced damage HaCaT cell model. Unfermented *Passiflora edulis* Sims peel water extract (PW) was used as the control, and the change of active substance content and in vitro antioxidant activity after the fermentation of *Passiflora edulis* Sims peel were determined to investigate the protective effects of *Passiflora edulis* Sims peel fermentation broth (PF) and PW on UVB-induced skin inflammation and skin barrier damage.

## 2. Materials and Methods

### 2.1. Materials and Reagents

The following materials and reagents were used: *Passiflora edulis* Sims peel, Yunnan, China; *Saccharomyces cerevisiae*, Culture Collection Center of the Beijing Institute of Food and Brewing, Beijing, China; Human immortalised epidermal cells (HaCaT), China Institute of Inspection and Quarantine; DMEM medium, Phosphate-Buffered Saline (PBS), fetal bovine serum, streptomycin, penicillin and 0.25% trypsin (including EDTA), GIBCO Life Technologies (CA, USA); folin reagent, Sinopharm Group (Beijing, China); Pyrogenic Gallic Acid, Sinopharm Chemical Reagent Co., Ltd. (Beijing, China); 1,1-diphenyl-2-trinitrophenylhydrazine (DPPH), Shanghai Maccai Biochemical Technology Co., Ltd. (Shanghai, China); fertilized chicken embryo of white Leghorn chicken, Beijing Guichuan Yashen Breeding Center (Beijing, China); defibrinated rabbit blood, Shanghai Yuanye Biotechnology Co., Ltd. (Shanghai, China); Cell Counting Kit-8 and total antioxidant capacity test kit (ABTS method), Biorigin Biotechnology Co., Ltd. (Beijing, China); BCA Protein Quantification Kit, Total Sugar Content Assay Kit, Solarbio Technology Co., Ltd. (Beijing, China); ROS, IL-1β, IL-8, TNF-α, HO-1, NQO1, KLK-7, AQP-3, FLG, Caspase 14, etc. assay kits, Nanjing Jiancheng Institute of Bioengineering (Nanjing, China), Cloud-Clone Corp. (Wuhan, China) Beyotime Biotechnology Co., Ltd. (Shanghai, China); microplate reader, cell incubator, Shanghai Yiheng Technology Co., Ltd.; 6-well plate, 96-well plate, Corning; Enzyme Labeler, Dickon (Shanghai) Trading Co., Ltd.; qPCR related kit, Thermo Fisher Scientific (Shanghai, China) Co., Ltd.

### 2.2. Preparation of PW and PF

Fresh *Passiflora edulis* Sims surface peel without bumps was taken, washed with deionized water, dried for 42 h at 60 °C and ground in a knife-mill.

Preparation of PW: The sample was dissolved in deionized water at a solid–liquid ratio of 2.5% and extracted in a water bath at 80 °C for 2 h. After standing to cool, it was centrifuged at 5000 rpm for 10 min, and the supernatant was taken to obtain *Passiflora edulis* Sims peel water extract.

The preparation of PF included a pretreatment that was the same as that for PW. After being extracted in a water bath at 80 °C for 2 h, it was sterilized at 115 °C for 20 min. After standing to cool, it was inserted into a 5% *Saccharomyces cerevisiae* bacteria solution, subjected to 28 °C air shaker fermentation for 48 h and centrifuged at 5000 rpm for 10 min, and the supernatant was taken to obtain *Passiflora edulis* Sims peel fermentation broth.

### 2.3. Determination of PW and PF Composition

Total Phenols: The total phenol content of PW and PF was determined by Forint phenol assay. A total of 4.43 mg of gallic acid standard was accurately weighed, the volume was fixed to 10 mL with distilled water, then 20, 40, 80, 120, 160 and 200 μL of the standard was pipetted into a 1.5 mL EP tube, and the volume fixed to 1 mL with distilled water. The standard group, blank group and sample group were set up, respectively. 100 μL of liquid was taken from each of the above EP tubes and put into six 1.5 mL EP tubes, and 100 μL of distilled water was added in turn and recorded as the standard group. The blank group consisted of 200 μL of distilled water. The sample group consisted of 100 μL of 1 mg/mL PW and PF fermentation solution and 100 μL of distilled water. For all groups, 50 μL of forinol reagent, 150 μL of 26.7% Na_2_CO_3_ solution and 600 μL of distilled water were added to make a total of 1 mL in the EP tube, then mixed and left at room temperature for 2 h. The absorbance was measured at 760 nm, the standard curve was plotted and the total phenolic content was calculated from the gallic acid standard curve.

Total Flavonoids: The total flavonoid content of PW and PF was determined by NaNO_2_-Al(NO_3_)_3_ assay. 1, 2, 3, 4 and 5 mL of rutin were added to six 10 mL test tubes, and 4, 3, 2, 1 and 0 mL of 30% ethanol solution were added to each of the six test tubes to form the standard group. The blank group included 5 mL of 30% ethanol solution. The sample group included 3 mL of 30% ethanol solution added to 2 mL of 1 mg/mL PW and PF. 1 mL was taken from each of the above groups, mixed with 0.3 mL of 5% NaNO_2_ solution and left for 6 min. Next, 0.3 mL of 10% Al(NO_3_)_3_ solution was added, mixed and left for 6 min. Then, 4 mL of 1 mol/L NaOH solution and 0.4 mL of distilled water were added and shaken well for 10 min. The absorbance was measured at a wavelength of 510 nm, the standard curve was plotted and the total flavonoid content was calculated using the rutin standard curve.

The total protein and total sugar content were ascertained with the BCA Protein Concentration Assay Kit and the Total Sugar Assay Kit, and the procedures were carried out according to the instructions to determine the total protein and total sugar content of 1 mg/mL PW and PF, respectively.

### 2.4. In Vitro Antioxidant Activity Assay

According to [18], the DPPH and hydroxyl radical scavenging rates of PF and PW were determined. The determination of total antioxidant capacity was determined with the ABTS and FRAP method total antioxidant capacity test kit, and the experimental procedure was carried out according to the reagent manufacturer’s instructions. The standard curve was drawn by Trolox and FeSO_4_·7H_2_O standard solution to determine the antioxidant capacity of PF and PW.

### 2.5. Cell Culture and Viability Experiment

After UVB damage to HaCaT cells, cell survival was examined by CCK-8 assay to investigate the protective effects of PF on damaged cells. HaCaT cells were cultured in flasks containing 10% fetal bovine serum and 1% penicillin-streptomycin DMEM medium and incubated in an incubator containing 5% CO_2_ at 37 °C for 2–3 days.

100 μL of HaCaT cell suspension (7 × 10^4^–1 × 10^5^ cells/mL) was added to each well of a 96-well plate and preincubated for 12 h. The culture was then discarded and washed twice with PBS, and 100 μL of sample was added to each well. Sample concentrations were 4, 2, 1, 0.5, 0.25 and 0.125 mg/mL. Serum-free DMEM was added to the control and blank wells and incubated for 24 h, and the culture solution was discarded. 10 μL CCK-8 was then added and incubated for a further 3 h, before the absorbance was measured at 450 nm and the cell viability calculated.

To model UVB cell damage, HaCaT cells were exposed to UVB at a dose of 40 mj/cm^2^ for 80 s using an ultraviolet phototherapy apparatus equipped with a 20W ozone-free UVB light. To investigate the protective effects of PF on HaCaT cells, the sample concentrations were 4, 2, 1, 0.5 and 0.25 mg/mL, the experimental method was the same as above and the absorbance was measured at 450 nm to determine the cell viability.

Cell survival (%) = (absorbance value of measurement wells—absorbance value of blank control)/(absorbance value of cell control—absorbance value of blank control) × 100%.

### 2.6. Measurement of Intracellular ROS Levels

HaCaT cells were inoculated in 6-well plates with 2 mL DMEM complete medium at a density of 5 × 10^5^ cells/well. Blank control group C, model group M and the sample group were established. After discarding the medium, 2 mL of serum-free DMEM was added to blank group C and model group M, and equal volumes of PW and PF were added to the sample group. After 24 h of incubation at 37 °C and 5% CO_2_, the medium was discarded, and a small amount of PBS (pH = 7.4) was added to cover the cells. The cells were then stimulated with UVB except for blank group C, replaced with serum-free DMEM and incubated for 12 h at 37 °C and 5% CO_2_. After discarding the cell culture medium, DCFH-DA 1:1000 diluted into serum-free medium, and 2 mL was added to each well. The cells were then incubated for 20 min at 37 °C and washed three times with serum-free DMEM to completely remove any DCFH-DA that had not entered the cells. The liquid was then discarded, and 1 mL of PBS was added to cover the cells. The intracellular ROS fluorescence content was observed, and the fluorescence intensity was recorded.

### 2.7. ELISA

Enzyme-linked immunosorbent assay (ELISA) provides the rapid and accurate detection of target enzyme activity and protein levels. The concentrations of inflammatory HaCaT cytokines TNF-α, IL-8 and IL-1β were measured by ELISA. HaCaT cells were inoculated in 6-well plates at a density of 5 × 10^5^ cells/well. After incubation at 37 °C and 5% CO_2_ for 24 h, the medium was decanted and the cells washed once with PBS, and 1 mL of PBS was added to each well to cover the adherent cells. 2 mL of PW and PF were added to the sample wells (blank controls were treated with serum-free DMEM) and incubated for 24 h. The medium was then discarded, and the cells were washed twice with PBS. HaCaT cells were exposed to UVB irradiation at a dose of 40 mj/cm^2^ for 80 s using an ultraviolet phototherapy apparatus equipped with a 20W ozone-free UVB lamp. The PBS was then discarded and serum-free DMEM was added and incubated for 24 h at 37 °C in a 5% CO_2_ incubator. 200 μL of lysis buffer was added and the cells were lysed with a cell scraper and centrifuged for 5 min to obtain the cell lysis supernatant. Inflammatory factors were measured according to the manufacturer’s operating instructions.

The antioxidant enzyme content and skin barrier-related factors were determined in accordance with the experimental methods described above. ELISA was used to determine the effects of PW and PF on the content of heme oxygenase-1 (HO-1), NAD(P)H quinone oxidoreductase 1 (NQO1), filamentous protein (FLG), aquaporins (AQP3), klikrein-7 (KLK-7) and caspase 14 in HaCaT cells after UVB damage, following the manufacturer’s instructions.

### 2.8. qRT-PCR

The blank group, model group and sample group were set up, and HaCaT cells were inoculated in 6-well plates at a density of 5 × 10^5^ cells/well for 24 h. TriQuick Total RNA Extraction Reagent was used to extract RNA from the HaCaT cells according to the instructions. The FastQuant cDNA kit was used to perform the cDNA first strand synthesis reaction. The cDNA obtained by further reverse transcription was examined using Fast Super EvaGreen^®^ qPCR Master Mix and qRT-PCR (QuantStudio 3, Thermoscientific, Shanghai, China). The primer sequences are shown in Table 1.

### 2.9. Safety Determination of PF

#### 2.9.1. Red Blood Cell Hemolysis Test

Using the Red Blood Cell Hemolysis Test, 3 mL of fresh defibrinated rabbit blood was added to 5 mL of PBS buffer and centrifuged at 3800 rpm for 5 min, and the yellow supernatant was discarded; this procedure was repeated three times. The precipitated cells were then diluted tenfold with PBS buffer. Next, 0.5 mL of cell suspension was added to 10 mL of distilled water and mixed, and its absorbance at 541 nm was measured at 0.5 ± 5%, which was the appropriate concentration of red blood cell suspension (RBC).

The RBC was mixed with different concentrations of sample at a ratio of 1:3, added to EP tubes, incubated for 60 min at 37 °C in a shaker and centrifuged at 10,000 rpm for 1 min, and the supernatant was then taken and recorded as A1. For the negative control, RBC mixed with PBS buffer at a ratio of 1:3 was added to EP tubes and recorded as A2. For completely haemolytic positive control, RBC mixed with deionized water at a ratio of 1:3 was added to EP tubes and recorded as A3. The absorbance values of each group were measured at 560 nm, and the haemolysis rate of the samples was calculated. Haemolysis rate = (A1 − A2)/(A3 − A2) × 100%.

#### 2.9.2. Chicken Embryo Chorioallantoic Membrane Test (HET-CAM)

Fresh eggs weighing 50–60 g were chosen. Incubation conditions included temperature 37.5 °C ± 0.5 °C, humidity 60–70%. The air chambers were positioned facing upwards and the cells were incubated for 9–11 days. Positive control (0.1 mol/L NaOH solution), negative control (0.9% NaCl solution) and sample groups (PF and PW) were set up, respectively. The eggshell was carefully peeled away with tweezers to reveal the surface of the chorioallantoic membrane (CAM). Next, 0.3 mL of the control and sample group solution was added, and the CAM was observed and recorded after 3 min for vascular damage in the form of bleeding, coagulation and vascular melting in six parallel sets of tests. The degree of eye irritation caused by the tasted substance was classified according to the IS values in Table 2. The calculation formula of the stimulus score is:

I = [(301 − sec *H*) × 5/300] + [(301 − sec *L*) × 7/300] + [(301 − sec *C*) × 9/300]

sec *H*: Average bleeding time of membrane;

sec *L*: Average time of vascular melting in membrane;

sec *C*: Average time of coagulation in membrane.

### 2.10. Statistical Analysis

Three separate experiments were carried out in which each sample was technically replicated and analysed three times. Excel was used for the experimental data statistics, and all values were expressed as mean ± standard deviation. Analytical plots were drawn using GraphPad Prism 8. T-test was used to determine the significance of differences between groups (^ns^ *p* > 0.05, * *p* < 0.05; ** *p* < 0.01, *** *p* < 0.001). When *p* < 0.05, the difference was considered statistically significant.

## 3. Results

### 3.1. Active Substance Content of PF and PW

**Figure 2:**
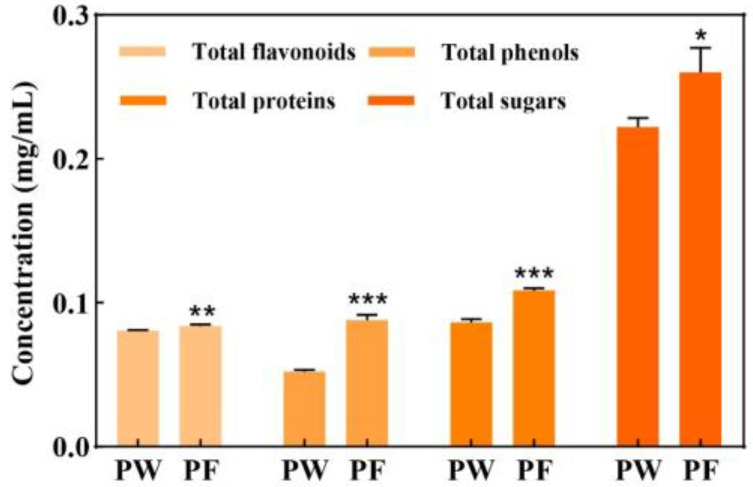
Active substance content of PF and PW. PW: *Passiflora edulis* Sims peel water extract. PF: *Passiflora edulis* Sims peel fermentation broth. Each value is expressed as mean ± SD (*n* = 3). (*: *p* < 0.05, **: *p* < 0.01, ***: *p* < 0.001, versus PW group).

The microbial fermentation method was used to ferment *Passiflora edulis* Sims peel by *Saccharomyces cerevisiae*. During the fermentation process, bacteria release enzymes which promote the release of the active substances in the peel and help to improve the efficacy and utilisation of *Passiflora edulis* Sims peel extract. In order to explore the advantages of PF, we analysed the total flavonoids, total phenols, total proteins and total sugars in PW and PF, as shown in Figure 2 and Table 3.

The contents of total flavonoids, total phenols, total protein and total sugars in PW and PF were determined, the histograms are shown in Figure 2, and the specific substance content are shown in Table 3. In the active substance content of PW, the content of total sugar was the highest and the content of total phenol was the lowest. The PW active substance content is all lower than PF. After fermentation treatment, the total phenolic content of *Passiflora edulis* Sims peel was significantly enhanced, and other active ingredients were also enhanced to varying degrees. The experimental results showed that there was no significant change in the content of flavonoids in PF compared to PW, but the latter could effectively increase the contents of total phenols, total proteins and total sugars.

### 3.2. In Vitro Antioxidant Activity of PF and PW

**Figure 3:**
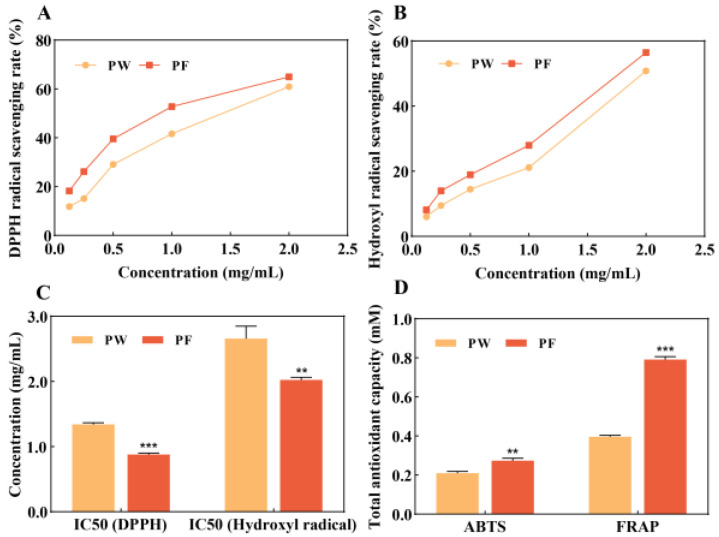
In vitro antioxidant capacity of PW and PF. (**A**): DPPH radical scavenging ability; (**B**) Hydroxyl radical scavenging ability; (**C**): Total antioxidant capacity (ABTS and FRAP methods). (**D**): PW: *Passiflora edulis* Sims peel water extract. PF: *Passiflora edulis* Sims peel fermentation broth. Each value is expressed as mean ± SD (*n* = 3). (**: *p* < 0.01, ***: *p* < 0.001, versus PW group).

Both metabolic and exogenous factors can lead to an increase in the level of ROS in the body, disrupting the oxidative balance and damaging the body at the cellular level. Antioxidant effects can be achieved by blocking the free radical reaction and reducing the accumulation of ROS [19]. When evaluating the in vitro antioxidant capacity of a substance, two or more free radicals are often selected to determine the maximum scavenging rate of the antioxidant substance due to the different mechanisms of action for scavenging free radicals. Meanwhile, the ABTS and FRAP methods are commonly used to assay total antioxidant capacity in vitro [20]. We determined the scavenging effects of PW and PF on DPPH, hydroxyl radicals and total antioxidant capacity, as shown in Figure 3.

In addition to measuring the antioxidant activity of PW and PF, we also measured the scavenging ability of resveratrol DPPH and hydroxyl radicals. The results showed that the scavenging rates of resveratrol DPPH and hydroxyl radicals at a concentration of 200 μg/mL were 63.42% and 74.4%, respectively.

The DPPH radical scavenging data showed that the samples had weak scavenging effects on DPPH radicals in the concentration range of 0.125–0.5 mg/mL, below 40%. The scavenging effect increased with the increase in sample concentration, reaching 64.96% for PF and 60.99% for PW at a concentration of 2 mg/mL. The hydroxyl radical scavenging assay showed that the samples had weak scavenging effects on hydroxyl radicals in the concentration range of 0.125–1 mg/mL, below 30%. When the concentration reached 2 mg/mL, the scavenging ability of PW and PF increased significantly, with the clearing rate of 56.46% for PF and 50.8% for PW.

The IC50 value, the half-inhibitory concentration, reflects the ability of an antioxidant to scavenge free radicals. The lower the IC50 value, the better the scavenging effects. The experimental data showed that PF and PW were more effective in scavenging DPPH free radicals.

The experimental results of the total antioxidant capacity ABTS and FRAP methods showed that PF had better total antioxidant activity than PW. PF achieved a scavenging rate of 0.27 mM Trolox equivalent for ABTS+. The reduction ability of PF on Fe^2+^ was 0.79 mM Trolox equivalent, 1.99 times that of PW.

### 3.3. Effects of PF and PW on HaCaT Cell Activity

**Figure 4:**
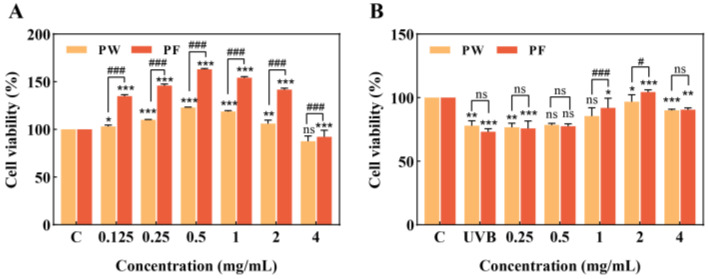
Effects of PW and PF on cell viability. (**A**): Effects of PW and PF on cell viability at different concentrations. (**B**): Effects of PW and PF on cell viability at different concentrations after UVB-induced damage. PW: *Passiflora edulis* Sims peel water extract. PF: *Passiflora edulis* Sims peel fermentation broth. Each value is expressed as mean ± SD (*n* = 3). (*: *p* < 0.05, **: *p* < 0.01, ***: *p* < 0.001, ns > 0.05, versus control group; #: *p* < 0.05, ###: *p* < 0.001, ns > 0.05, versus PW group).

The effects of PF and PW on the viability of HaCaT cells were investigated. Figure 4A shows that a low concentration of PF was non-toxic to HaCaT cells, the survival rate of the cells was above 80%, the effects were proliferative and the effects of PF were more significant than those of PW.

A UVB cell damage model was established in which HaCaT cells were exposed to UVB irradiation at a dose of 40 mj/cm^2^ for 80 s using an ultraviolet phototherapy apparatus equipped with a 20W ozone-free UVB light. As shown in Figure 4B, the cell survival rate of HaCaT cells after UVB damage was lower than 80% of that of unirradiated damaged cells. After treatment with PW and PF, the samples at 1, 2 and 4 mg/mL concentrations were protective for HaCaT cells, among which the 2 mg/mL sample had a significant protective effect on the cells with cell viability close to that of unirradiated damaged cells, and PF had a better effect than PW. However, lower concentrations of the sample were less protective for HaCaT cells. Based on cell viability and status, we selected 2 mg/mL of PF and PW for subsequent experiments.

### 3.4. ROS Inhibition and Antioxidant Capacity of PF and PW

Acute damage from UVB irradiation induces excessive ROS production in cells, which disrupts the balance between oxidative and antioxidant action in the body and triggers oxidative stress, resulting in an inflammatory response in cells [15].

HO-1 is a stress response protein [21], which is mainly expressed in skin cells and is closely related to epidermal differentiation [22]. NQO1 is a detoxification enzyme, and the increase of its activity can protect against the development of skin carcinogenesis [23]. HO-1 and NQO1 belong to the Phase II (antioxidant and detoxification) enzymes which are important defense systems against harmful stressors. They can fight against ROS and participate in protecting cells from oxidative stress [24].

**Figure 5:**
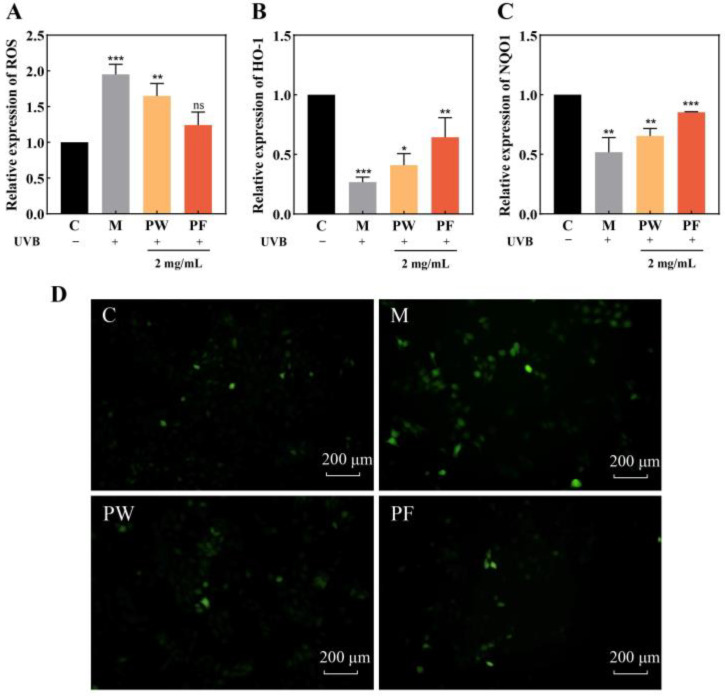
Effects of PF and PW on the activity of ROS, antioxidant enzymes and detoxification enzymes in HaCaT cells. (**A**): Cell fluorescence intensity value. (**B**): Effects of 2 mg/mL PF and PW on activity of HO-1 antioxidant enzymes in HaCaT cells. (**C**): Effects of 2 mg/mL PF and PW on activity of NQO1 detoxification enzymes in HaCaT cells. (**D**): Cell ROS fluorescence intensity. PW: *Passiflora edulis* Sims peel water extract. PF: *Passiflora edulis* Sims peel fermentation broth. C: control group. M: UVB damage model group. Each value is expressed as mean ± SD (*n* = 3). (*: *p* < 0.05, **: *p* < 0.01, ***: *p* < 0.001, ns > 0.05, versus control group).

ROS was detected in HaCaT cells using a DCFH-DA fluorescent probe. The scavenging effects of 2 mg/mL PF and PW on ROS are shown in Figure 5A,D. Blank group C without UVB irradiation had low active oxygen content, less fluorescence and weaker intensity. After UVB damage, the ROS content of the model group was higher, the green fluorescence was significantly increased and the fluorescence intensity was enhanced. After PF and PW protection, the cells were irradiated with UVB. Compared with the model group, the ROS content decreased significantly, the fluorescence amount was less and the fluorescence intensity was clearly weakened. Compared with PW, the ROS content of cells treated with PF was lower, and the scavenging effect was better. Studies have shown that PF and PW can effectively reduce ROS content and have significant ROS scavenging effects.

Figure 5B,C show the effect of PF and PW on the cellular content of HO-1 and NQO1. UVB irradiation resulted in a significant decrease in intracellular HO-1 and NQO1 content compared to the blank control group. The loss of HO-1 and NQO1 protein content in HaCaT cells was less after PF and PW interventions. In particular, the HO-1 content in cells after PF treatment was twice as high as that in the model group. In conclusion, PF and PW had significant protective effects on cellular oxidative stress damage after UVB irradiation, with PF showing effects superior to those of PW.

### 3.5. Effects of PF and PW on Cellular Inflammatory Factors and Gene Expression

**Figure 6:**
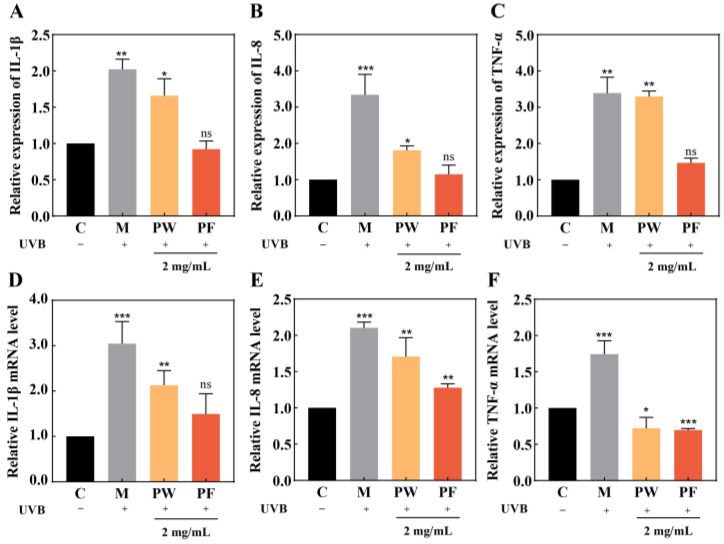
Effects of 2 mg/mL of PF and PW on inflammatory factor activity and relative mRNA expression in HaCaT cells. (**A**,**D**) IL-1β activity and mRNA expression levels; (**B**,**E**) IL-8 activity and mRNA expression levels; (**C**,**F**) TNF-α activity and mRNA expression levels. PW: *Passiflora edulis* Sims peel water extract. PF: *Passiflora edulis* Sims peel fermentation broth. C: control group. M: UVB damage model group. Each value is expressed as mean ± SD (*n* = 3). (*: *p* < 0.05, **: *p* < 0.01, ***: *p* < 0.001, ns > 0.05, versus control group).

Several studies have shown that UVB damage in cells can lead to the expression of a variety of inflammatory mediators including interleukin-1β (IL-1β), interleukin-8 (IL-8) and tumor necrosis factor-α (TNF-α) [25,26,27]. We measured the protein levels of these three inflammatory factors and their gene expression using qPCR and ELISA. The relative expressions are shown in Figure 6. Compared with the blank group, the secretion of inflammatory factors was significantly increased in the model group after UVB irradiation damage, the contents of IL-1β, IL-8 and TNF-α were elevated in HaCaT cells, and their mRNA expressions were significantly up-regulated. Cells treated with PW and PF showed a smaller increase in inflammatory factor protein content, which was much lower than that of the model group. PF was more effective in inhibiting the release of pro-inflammatory factors.

### 3.6. Effects of PF and PW on Skin Barrier-Related Factors and Gene Expression

Studies have shown that human kallikrein-related peptidases (KLKs) may be associated with various types of skin inflammation and typically function to cause physiological flaking [28], with KLK-7 active in the damaged surface layer of human skin and thought to cause skin flaking through the direct degradation of keratin bridging granules [29,30]. FLG is an important structural protein, which is a source of moisturising factors for epidermal cells. The aquaporins (AQPs) are transmembrane transporter proteins associated with the permeability of water molecules in keratin-forming cells; they transport water and glycerol and are thought to be a key factor in maintaining skin hydration [31], while AQP3 is the main AQP in the human epidermis. Caspase 14 is a protease that is mainly activated during the keratinisation process of keratin-forming cells and helps epidermal cells to prevent water loss, and the dysregulated expression of caspase 14 is associated with impaired skin barrier formation [32]. The maintenance of a healthy epidermal cell structure is closely related to the normal expression of KLK-7, AQP3, FLG and Caspase 14 proteins.

**Figure 7:**
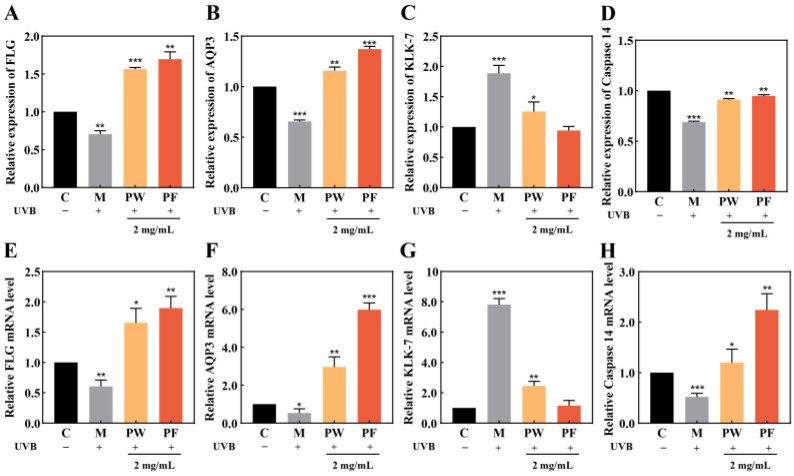
Effects of 2 mg/mL PF on activity and mRNA expression of skin barrier-related factors in HaCaT cells. (**A**,**E**) FLG protein activity and mRNA expression level; (**B**,**F**) AQP3 protein activity and mRNA expression level; (**C**,**G**) KLK-7 enzyme activity and mRNA expression level; (**D**,**H**) Caspase 14 enzyme activity and mRNA expression level. PW: *Passiflora edulis* Sims peel water extract. PF: *Passiflora edulis* Sims peel fermentation broth. C: control group. M: UVB damage model group. Each value is expressed as mean ± SD (*n* = 3). (*: *p* < 0.05, **: *p* < 0.01, ***: *p* < 0.001, versus PW group).

qPCR and ELISA were used to detect the expression of skin barrier-related proteins and their genes in HaCaT cells. As shown in Figure 7, the relative expression of KLK-7 was significantly increased, and the relative expressions of AQP3, FLG and Caspase 14 were significantly decreased in the model group after damage by UVB irradiation compared with the blank group. Compared with the model group, the relative expression of KLK-7 was significantly lower, and the relative expressions of AQP3, FLG and Caspase 14 were significantly higher in cells that were first treated with PW and PF before UVB damage, with PF having a more significant effect. The results showed that PW and PF could effectively regulate the content of skin barrier-related factors and contribute to their normal gene expression.

### 3.7. Regulation of PI3K/AKT/mTOR Signalling Pathway

AKT, also known as protein kinase B (PKB), is a downstream mediator of phosphatidylinositide 3 kinase (PI3K). PI3K, AKT and mammalian target of rapamycin (mTOR) constitute the PI3K/AKT/mTOR signalling pathway, which regulates several biological processes such as autophagy, apoptosis, survival and anti-inflammation and is closely related to cellular oxidative stress [33].

**Figure 8:**
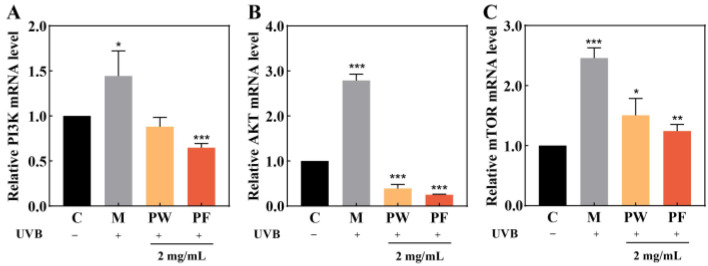
Effects of 2 mg/mL of PF on cellular PI3K/AKT/mTOR pathway gene expression. (**A**) Relative expression of PI3K in HaCaT cells; (**B**) Relative expression of AKT mRNA in HaCaT cells, (**C**) Relative expression of mTOR mRNA in HaCaT cells. PW: *Passiflora edulis* Sims aqueous extract. PF: *Passiflora edulis* Sims peel fermentation broth. C: control group. M: UVB damage model group. Each value is expressed as mean ± SD (*n* = 3). (*: *p* < 0.05, **: *p* < 0.01, ***: *p* < 0.001, versus PW group).

To further investigate the protective mechanism of PF against HaCaT cell damage induced by UVB irradiation, we used qPCR to detect the expressions of PI3K, AKT and mTOR genes in HaCaT cells. As shown in Figure 8, the expressions of related protein genes were significantly increased, and the PI3K/AKT/mTOR signalling pathway was activated after the UVB irradiation of cells compared to the blank group. The PI3K/AKT/mTOR signalling pathway was significantly inhibited in cells protected by PW and PF after UVB irradiation, with PF significantly more effective than PW.

### 3.8. Safety of PF and PW

**Figure 9:**
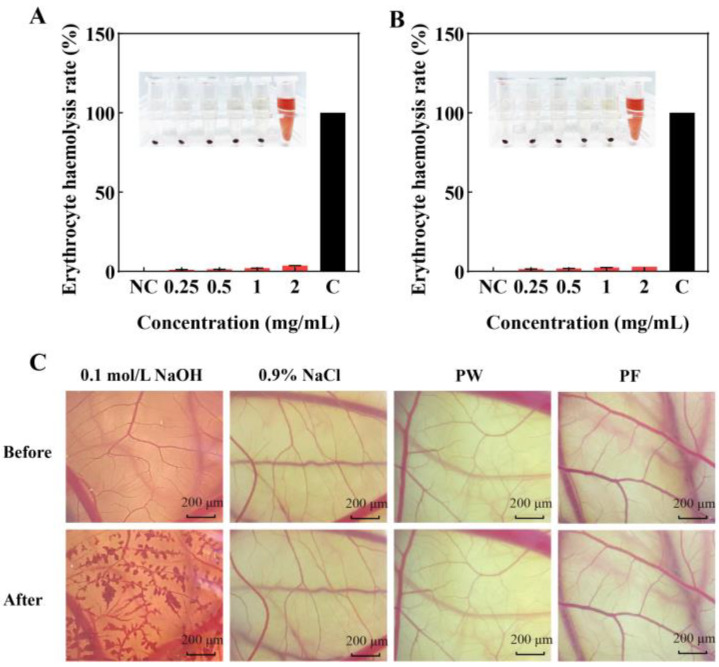
Safety of PF and PW: (**A**) Erythrocyte hemolysis rates for different concentrations of PW. (**B**) Erythrocyte hemolysis rates for different concentrations of PF. (**C**) Vascular presentation and response plots before and after representative CAM spiking for PF and PW. PW: *Passiflora edulis* Sims peel water extract. PF: *Passiflora edulis* Sims peel fermentation broth. NC: Negative control. C: Complete hemolysis control group. Each value is expressed as mean ± SD (*n* = 3).

The Erythrocyte Haemolysis test and Chicken Embryo Chorioallantoic Membrane test are classic safety tests for checking the irritation of samples [34]. Figure 9A,B show the histogram of the hemolysis rates of PW and PF at different concentrations. Sample concentrations from left to right are negative control, 0.25, 0.5, 1 and 2 mg/mL of sample solution and complete hemolysis control. Complete hemolysis control showed significant erythrocyte rupture and lysis with a 100% hemolysis rate. The erythrocyte haemolysis rates of PW and PF were less than 3% at sample concentrations lower than 2 mg/mL. The experiments showed that PW and PF had low degrees of erythrocyte membrane rupture and lysis, no irritation and good safety.

Figure 9C shows the stimulation effects on blood vessels before and after adding the sample. The stimulation score of the complete hemolysis control group was 18.93, blood vessel rupture was obvious and stimulation was strong. The stimulation scores of PW and PF were 0.09 and 0.07, respectively, with no hemolysis, no stimulation and excellent safety.

## 4. Discussion

The skin is the largest organ of the body, and its most important function is to act as a barrier between the external and internal environments. The skin barrier both reduces the skin’s internal loss of moisture and protects it from harmful external environmental agents. There are many categories of skin barrier, of which the physical barrier function is mainly provided by the stratum corneum, which has a certain self-regulatory effect and is the basis for maintaining the normal physiological activity of the skin [35]. Healthy skin is imbalanced in its ability to regulate itself in response to external stimuli and is unable to renew and repair itself in time. However, exposure to ultraviolet radiation, allergens and toxic chemical irritants can cause acute inflammatory damage to the skin [36], leading to the disruption of the skin barrier function. The skin may appear dry, flaky and itchy, which can lead to inflammatory skin diseases such as atopic dermatitis and psoriasis.

*Passiflora edulis* Sims peel has been shown to be rich in polyphenols, flavonoids, polysaccharides and other active substances with excellent antioxidant and inflammation-inhibiting properties, and microbial fermentation technology allows for the greater release of the active ingredients in the plant.

In this study, microbial fermentation techniques were used to treat *Passiflora edulis* Sims peel with *Saccharomyces cerevisiae* and examine the differences in PW and PF active substance content and in vitro free radical scavenging capacity. Studies have shown that fermentation can effectively increase the contents of total sugars, total phenols and total proteins in *Passiflora edulis* Sims peel, probably because the fermentation treatment of the peel is accompanied by changes in structure or molecular weight, thereby increasing its active substance content, and PF has a more significant free radical scavenging capacity than PW.

Previous studies have shown that *Passiflora edulis* Sims peel has significant anti-inflammatory effects, effectively inhibiting the secretion of cellular inflammatory factors and down-regulating related genes [5]. However, the role of *Passiflora edulis* Sims peel in regulating skin inflammation and maintaining the skin barrier function, and the mechanism of its protective effect on UVB irradiation, have not been confirmed. Keratinocytes are considered a typical cell model for studying skin barrier problems and a common cell model for exploring the mechanism of UVB radiation-induced cellular inflammatory response [37]. Therefore, we established a UVB-induced HaCaT cell damage model to explore the protective role and mechanism of PW and PF in the protection of the skin barrier and prevention of inflammatory damage caused by UVB irradiation. The experimental results showed that both PW and PF at low concentrations had a proliferative effect on HaCaT cells, with PF having a more significant proliferative effect. After UVB irradiation damage, cell viability was significantly down-regulated, and intracellular ROS levels increased. In terms of antioxidation, after PF treatment, the cells produced an antioxidant response by activating HO-1 and NQO1 proteins to protect themselves from ROS damage, and the level of intracellular ROS was significantly reduced.

Excessive UVB irradiation can cause the excessive secretion and up-regulation of inflammatory signalling factors such as TNF-α, IL-1β and IL-8 in keratinocytes [15], leading to skin inflammation, which in turn affects the normal function of the skin barrier, leading to skin homeostasis imbalance, dry and red appearance, and desquamation [16,17]. In the epidermis, KLK-7 [38], FLG, caspase 14 [39] and AQP3 [40] are proteins closely related to the skin barrier that can help the keratinocytes maintain good skin barrier function. The abnormal activation of KLK-7 affects the barrier function of the stratum corneum [28]. FLG is an important structural protein that can be decomposed into free amino acids and is the main source of skin moisturizing factors [41]. Studies have shown that caspase 14 can promote the catabolism of FLG and contribute to the synthesis of intercellular moisturizing factors [42]. AQP3 is a water/glycerol transporter in the skin. The abnormal decrease of AQP3 expression in the epidermis is an important factor leading to skin diseases such as psoriasis and specific dermatitis [43]. We have demonstrated that PF and PW can protect human epidermal cells; inhibit the expression of KLK-7; increase the protein content and expression level of FLG, AQP3 and caspase 14 in cells; effectively prevent cell detachment caused by UVB irradiation; increase the content of structural proteins in the epidermis and the supplemental level of intercellular moisturizing factors; enhance the skin’s defense against UVB-induced epidermal damage; maintain the normal function of the skin barrier and protect cells from UVB-induced irradiation damage.

In this study, the protective effects of PF and PW against UVB irradiation damage were confirmed by measuring the levels of ROS, antioxidant enzymes, inflammatory factors and skin barrier-related factors. To further investigate the protective mechanisms of PF and PW on skin inflammation and the skin barrier, we examined the effects of PF and PW on the transcriptional levels of key genes in the PI3K/AKT/mTOR signalling pathway.

The activation of ROS leads to the phosphorylation of the PI3K/AKT/mTOR pathway [44]. A large number of studies have shown that the PI3K/AKT/mTOR signalling pathway can regulate apoptosis [45], anti-inflammation [46], anti-cancer [47] and other biological processes. The inhibition of ROS production can effectively inhibit the activation of the PI3K/AKT/mTOR signalling pathway, thereby exerting anti-inflammatory activity and minimizing the occurrence of inflammatory diseases [48]. Studies have shown that UVB irradiation can activate the up-regulation of the PI3K/AKT/mTOR signalling pathway in keratinocytes [49,50], in which the high activation of AKT/mTOR will increase the content of inflammatory factors in cells, leading to inflammatory skin diseases [51]. In this study, HaCaT cells were treated with PF to detect the expression of related genes. The results showed that PF and PW could protect cells from the excessive accumulation of ROS caused by UVB irradiation and inhibit the activation of the PI3K/AKT/mTOR signalling pathway.

UVB damage to cells is multifaceted, and there are some limitations in this experiment. Other studies have shown that excessive UVB irradiation causes cellular DNA damage and mitochondrial dysfunction in addition to intracellular ROS accumulatio [52,53]. This is a direction worth exploring in depth in subsequent experiments, which can verify the protective effect of PW and PF on photodamage of HaCaT cells in many ways.

## 5. Conclusions

In this study, the fermentation of *Passiflora edulis* Sims peel was carried out by microbial fermentation technology using *Saccharomyces cerevisiae*. This process enhances the content of active substances in the peel and improves its free radical scavenging ability and better antioxidant properties. The Erythrocyte Haemolysis test and Chicken Embryo Chorioallantoic Membrane test demonstrate that low concentrations of *Passiflora edulis* Sims peel have no irritation. Through biochemical, cellular and cellular molecular analyses, it was shown that *Passiflora edulis* Sims peel can protect the skin from oxidative stress-induced inflammation and skin barrier function damage, inhibit the release of pro-inflammatory factors and regulate the expression of skin barrier-related proteins by inhibiting the activation of the PI3K/AKT/mTOR signalling pathway. However, further research is required to prove this theory in multiple dimensions, such as animal experiments, histology and other techniques. In conclusion, PF has the potential to be developed and used as a functional protective agent to reduce UV damage to the skin, improve the utilisation of *Passiflora edulis* Sims peels and make more efficient use of natural plant resources.

## Figures and Tables

**Table 1:** Primers.

Primers	Direction	Primer Sequence (5′–3′)
β-actin	F	TGGCACCCAGCACAATGAA
	R	CTAAGTCATAGTCCGCCTAGAAGCA
IL-1β	F	AACCTCTTCGAGGCACAAGG
	R	GTCCTGGAAGGAGCACTTCAT
IL-8	F	GGAGAAGTTTTTGAAGAGGGCTG
	R	ACAGACCCACACAATACATGAAG
TNF-α	F	TCTCCTTCCTGATCGTGGCA
	R	CAGCTTGAGGGTTTGCTACAAC
KLK-7	F	TCAGATCCTCTCGAGCCCAG
	R	CAGGTGCACGGTGTACTCAT
FLG	F	TGAGGCATACCCAGAGGACT
	R	CTGTATCGCGGTGAGAGGAT
AQP3	F	CTTCTTTGACCAGGACCGGC
	R	GGGCCAGCTTCACATTCTCT
Caspase 14	F	ATCCAGACCCTGGTGGATGT
	R	GATACAGCCGTTTCCGGAGG
PI3K	F	TCCCTTCGATAAGAGTCGAGG
	R	GCAGTCTTGTCGCAAAGTCC
AKT	F	AAGTCATCGTGGCCAAGGAC
	R	ACAGGTGGAAGAACAGCTCG
mTOR	F	CTTAGAGGACAGCGGGGAAG
	R	TCCTTTAATATTCGCGCGGC

**Table 2:** Results of irritation evaluation score.

Stimulus Score	Classification
IS < 1	No irritation
1 ≤ IS < 5	Mid irritation
5 ≤ IS < 9	Moderate irritation
IS ≥ 9	Strong irritation

**Table 3:** Active substance content of PW and PF.

Sample	Total Flavonoids (mg/mL)	Total Phenols (mg/mL)	Total Proteins (mg/mL)	Total Sugars (mg/mL)
PW	0.080 ± 0.001	0.052 ± 0.003	0.086 ± 0.004	0.109 ± 0.003
PF	0.084 ± 0.002	0.088 ± 0.007	0.122 ± 0.012	0.160 ± 0.031

## Data Availability

The data are available from the corresponding author upon reasonable request.
